# Successful conservative management of urinary tract rupture in dogs and cats: 52 cases (2003‐2024)

**DOI:** 10.1111/jsap.13882

**Published:** 2025-06-01

**Authors:** C. S. L. Toh, M. Rossanese, S. D. Cook

**Affiliations:** ^1^ Royal Veterinary College University of London Hatfield UK

## Abstract

**Objectives:**

To describe the successful conservative management of urinary tract ruptures in dogs and cats.

**Materials and Methods:**

Medical records of a hospital between 2003 and 2024 were reviewed to identify dogs and cats with urinary tract rupture. Cases were included if they were successfully managed conservatively (including only procedures that did not directly address the site of rupture). Data recorded included signalment, cause and location of rupture, method and duration of urinary diversion, outcome and complications.

**Results:**

Fifty‐two cases (40 cats and 12 dogs) were included. The most common causes of rupture were trauma associated with urethral obstruction and catheterisation (18), cystocentesis (17) and external trauma (8). The most common sites of rupture were the urethra (20 cats and five dogs) and urinary bladder (15 cats and four dogs). Bladder ruptures were most commonly managed with urethral catheters and/or peritoneal drains, while urethral ruptures were most commonly managed with urethral catheters and/or cystostomy tubes. The median (range) time to resolution of urine leakage documented on imaging was 3 (1 to 6) days for bladder ruptures and 6.5 (3 to 28) days for urethral ruptures. Radiographic evidence of urethral narrowing was documented in 11/25 cases with urethral tears at a median (range) of 12 (4 to 28) days post‐rupture. Urine culture was performed in 22/52 cases with urinary tract ruptures and was positive in 14 cases.

**Clinical Significance:**

Conservative management can be considered in both iatrogenic and traumatic urinary tract ruptures. The risk of urethral strictures and urinary tract infections should be considered when electing for conservative management of urinary tract ruptures.

## INTRODUCTION

Urinary tract rupture in dogs and cats can result from various insults, including external trauma and iatrogenic damage. This condition leads to the accumulation of urine in the peritoneal cavity, retroperitoneal cavity or subcutaneous tissues, causing local tissue damage and electrolyte imbalances such as life‐threatening hyperkalaemia (Robakiewicz & Halfacree, 2023; Stafford & Bartges, 2013).

Management of patients with urinary tract rupture involves initial haemodynamic stabilisation and correction of electrolyte imbalances where possible, followed by addressing the rupture itself. Treatment options include primary repair of the rupture, salvage procedures to remove damaged portions of the urinary tract (*e.g*. ureteronephrectomy), permanent urinary diversion (such as urethrostomy) or temporary urinary diversion as part of conservative management to allow second intention healing (Addison et al., 2014; Robakiewicz & Halfacree, 2023; Stafford & Bartges, 2013).

Multiple methods of conservative management for urethral tears and uroperitoneum have been described, including retrograde urethral catheterisation (Meige et al., 2008; Stafford & Bartges, 2013), antegrade urethral catheterisation (Holmes et al., 2012; Meige et al., 2008), cystostomy tube placement (Anderson et al., 2006; Beck et al., 2007; Nurra et al., 2022) and peritoneal catheter placement (Crosby et al., 2023).

The choice of management depends on the location and severity of the urinary tract rupture, cardiovascular stability of the patient and clinician preference (Grimes et al., 2018; Meige et al., 2008; Robakiewicz & Halfacree, 2023; Stafford & Bartges, 2013). Conservative management with urinary diversion may be preferred over primary surgical repair or permanent surgical urinary diversion, particularly in unstable patients, as it is relatively simple to perform, is less invasive and usually requires less procedural time (Crosby et al., 2023; Holmes et al., 2012; Meige et al., 2008).

The majority of cases with urinary tract rupture in the published literature appear to have been managed surgically, with studies reporting 75% to 95% of cats undergoing primary closure of the bladder defect, urethrostomy or urethral resection and anastomosis (Addison et al., 2014; Aumann et al., 1998; Hornsey et al., 2021) and 86% of dogs undergoing primary closure of the defect (Grimes et al., 2018). There is a relative paucity of literature on the use or success of conservative management. The aim of this study was to describe the successful conservative management of urinary tract rupture in dogs and cats, detailing their aetiology, clinical findings, method and duration of urinary diversion, outcomes and associated complications.

## MATERIALS AND METHODS

### Study design

The electronic medical records of the Queen Mother Hospital for Animals at the Royal Veterinary College between January 1, 2003 and May 12, 2024 were retrospectively searched on May 19, 2024 to identify dogs and cats with urinary tract rupture, using the following search terms: “urinary tract rupture”, “kidney rupture”, “ureteral rupture”, “ureteral tear”, “bladder rupture”, “bladder tear”, “bladder injury”, “urethral rupture”, “urethral tear”, “urethral injury”, “uroabdomen”, “uroperitoneum” and “uroretroperitoneum”. Cases were then assessed for inclusion.

### Inclusion criteria

Urinary tract rupture must have been diagnosed based on diagnostic imaging or biochemical comparison of cavitary effusion with peripheral blood. An effusion to blood creatinine ratio of more than 2:1 or an effusion to blood potassium ratio of more than 1.4:1 for dogs and more than 1.9:1 for cats was considered diagnostic of urinary tract rupture (Aumann et al., 1998; Schmiedt et al., 2001). Additional cases without biochemical analysis of abdominal effusion or diagnostic imaging consistent with urinary tract rupture were also eligible for inclusion if urinary tract rupture was highly suspected based on critical appraisal of the case records by all authors (*e.g*. development of a new peritoneal effusion and new progressive azotaemia after cystocentesis, without suspicion of haemoabdomen).

Cases were included if they were successfully managed conservatively, where conservative management was defined as involving only procedures that do not address the site of rupture directly. Success was defined as there being no further urine leakage or resolution of free cavitary fluid, as documented on diagnostic imaging or based on clinical resolution.

Cases were excluded if urinary tract rupture could not be confirmed based on the available medical records, if cases underwent surgical repair of the urinary tract rupture, if death occurred prior to the resolution of urinary tract rupture or if medical records were incomplete.

### Data extracted

Data recorded included signalment, cause of rupture, location of rupture, method of diagnosis, presence or absence of free cavitary fluid, evidence of urine leakage into subcutaneous tissues, plasma creatinine and potassium at the time of rupture, method and duration of urinary diversion, basis for determining success of conservative management, time to resolution of urinary tract rupture if documented by diagnostic imaging, duration of hospitalisation, duration of available follow‐up and the development of associated complications (*i.e*. strictures in cases of urethral or ureteral tears) or a positive bacterial culture during hospitalisation or available follow‐up.

Causes of rupture were categorised into trauma associated with urethral obstruction and catheterisation, cystocentesis, recent urinary tract surgery, external trauma or other. Locations of rupture were divided into the kidney, the ureter, the urinary bladder or the urethra. The location of rupture was either determined based on clinical suspicion or confirmed on diagnostic imaging. If urinary tract surgery was recently performed, the location of rupture was suspected to be from the surgical site unless documented otherwise on diagnostic imaging. If cystocentesis had been performed and free peritoneal fluid subsequently developed, the location of rupture was presumed to be the bladder. The method of diagnosis was recorded as diagnostic imaging, fluid analysis or appraisal of clinical records.

Cases were considered to have free fluid present if there was free fluid present within the peritoneal cavity or retroperitoneal space on ultrasonography. Cases were considered to have urine leakage into subcutaneous tissues if there was swelling of the soft tissues adjacent to the length of the urinary tract on physical examination. Azotaemia and hyperkalaemia were defined as elevations in creatinine and potassium levels above the laboratory analyser's specified reference interval. The method of urinary diversion was recorded as one or more of the following: urethral catheter, abdominocentesis, peritoneal drain or cystostomy tube. It was also recorded how these were placed [*i.e. via* minimally invasive methods (*e.g*. percutaneously) or surgically]. The duration of urinary diversion was defined as the number of days that the chosen device for urinary diversion was left indwelling, while the time to resolution of urinary tract rupture was defined as the number of days between diagnosis of urinary tract rupture and resolution of free abdominal fluid or urine leakage as documented on diagnostic imaging.

Follow‐up information was obtained from the available medical records only. Medical records were inspected for the development of urethral strictures (clinical signs referable to stricture formation and confirmed stricture on positive contrast radiography or endoscopy) and the presence of a positive bacterial culture of urine during the period of urinary diversion or within 48 hours of removal of the device used for urinary diversion. Patients with pre‐existing urinary tract infections were excluded from this analysis.

### Statistical analysis

Continuous data were assessed for normality using the Shapiro–Wilk test. Normally distributed data were reported as mean ± standard deviation, while non‐normally distributed data were reported as median and range (minimum to maximum). Categorical data were reported as number (*n*) and percentage (%) where appropriate.

## RESULTS

### Patient inclusion

The medical record search identified 875 dogs and cats. Of these cases, 478 cases were excluded as they did not have urinary tract rupture, 271 cases were excluded as they underwent surgical management, 62 cases were excluded as they were euthanased or went into cardiopulmonary arrest prior to management or resolution of their urinary tract rupture and 12 cases were excluded as they had incomplete medical records precluding confirmation of urinary tract rupture or their outcomes. Fifty‐two cases (40 cats and 12 dogs) were ultimately included in this study.

### Signalment

Cat breeds included 34 domestic shorthair or domestic longhair cats, three British shorthair cats and one each of Burmese, munchkin and Turkish Van cats. Dog breeds included four crossbreeds, two golden retrievers and one each of Border collie, cocker spaniel, English bulldog, Jack Russell terrier, Rottweiler and West Highland white terrier. The median age was 68.5 months (range, 6 to 154) for cats and 50.5 months (range, 5 to 199) for dogs. Among the 40 cats, 35 were male and five were female; all 40 were neutered. Among the 12 dogs, seven were male (two entire and five neutered) and five were female (one entire and four neutered).

### Cause and location of rupture

Causes of urinary tract rupture in cats included trauma associated with urethral obstruction and catheterisation (*n* = 17), cystocentesis (*n* = 14), external trauma (*n* = 5), recent urinary tract surgery (*n* = 3) and others (*n* = 1). In dogs, the causes included cystocentesis (*n* = 3), external trauma (*n* = 3), recent urinary tract surgery (*n* = 3), trauma associated with urethral obstruction and catheterisation (*n* = 1) and others (*n* = 2). Of the 17 cases of urinary tract rupture secondary to cystocentesis, all were urinary bladder ruptures. In all, 13 of these 17 cases had concurrent urethral obstruction due to feline lower urinary tract disease (*n* = 8), urolithiasis (*n* = 2), a urethral stricture (*n* = 1), a urethral mass (*n* = 1) and a sublumbar mass (*n* = 1).

Location of urinary tract rupture included the kidney in one cat and two dogs (confirmed on imaging in all three cases), the ureter in one cat and one dog, the urinary bladder in 15 cats and four dogs (confirmed on imaging in four cases), the urethra in 20 cats and five dogs (confirmed on imaging in 24 cases) and an unclear location in three cats. Table 1 details the types of urinary tract ruptures (categorised based on cause and location), which were successfully managed conservatively.

**Table 1:** Causes and sites of urinary tract rupture that were successfully managed conservatively

Cause of rupture	Kidney (*n* = 3)	Ureter (*n* = 2)	Bladder (*n* = 19)	Urethra (*n* = 25)	Unknown (*n* = 3)
Trauma associated with urethral obstruction and catheterisation				16 cats 1 dog	1 cat
Cystocentesis			14 cats 3 dogs		
External trauma	2 dogs			4 cats 1 dog	1 cat
Recent urinary tract surgery		1 cat 1 dog	1 cat	2 dogs	1 cat
Others	1 cat		1 dog	1 dog	

### Method of diagnosis

Urinary tract rupture was diagnosed based on diagnostic imaging alone in 29 cases, fluid analysis alone in 18 cases, a combination of imaging and fluid analysis in three cases and appraisal of clinical records in two cases. Of the 29 cases diagnosed based on imaging alone, 26 cases were diagnosed based on radiographs with contrast, two cases were diagnosed based on CT with contrast and one was diagnosed based on plain radiographs. Of the 18 cases diagnosed based on fluid analysis alone, the exact cavitary effusion to blood creatinine or potassium ratios was not available for review in nine cases, but the effusions were recorded as being consistent with uroabdomen by the attending clinician, and appraisal of the clinical records by the authors was also in agreement.

### Clinical findings

Point‐of‐care ultrasonography was performed in 44 out of 52 patients. This identified free fluid within the peritoneal or retroperitoneal cavity in 29 patients. This included all 21 out of 21 patients with tears involving the kidney or bladder, 6 out of 21 patients with urethral tears and two patients with unclear sites of rupture.

Urine leakage into the subcutaneous tissues was apparent in 6 out of 52 patients; all six patients had urethral tears. These patients had swelling of the scrotum, prepuce, perineum and/or pelvic limbs, with abscessation of a pelvic limb in one cat.

At the time of rupture diagnosis, 29 cats and four dogs were azotaemic, four cats and seven dogs were non‐azotaemic, while creatinine levels were unknown in the remaining cases (seven cats and one dog). Seventeen cats and two dogs were hyperkalaemic at the time of rupture diagnosis, 14 cats and nine dogs were normokalaemic, while potassium levels were unknown in the remaining nine cats and one dog.

### Methods of urinary diversion of urinary tract ruptures

Table 2 details the methods of urinary diversion used based on the site of urinary tract rupture.

**Table 2:** Methods of urinary diversion based on the site of urinary tract rupture

	Kidney (*n* = 3)	Ureter (*n* = 2)	Bladder (*n* = 19)	Urethra (*n* = 25)	Unknown (*n* = 3)	Overall median indwelling duration (range) (days)
None (*n* = 3)	2 dogs			1 cat		–
Peritoneal drain (*n* = 12)	1 cat	1 dog 1 cat	6 cats 2 dogs		1 cat	2 (1 to 7)
Urethral catheter (*n* = 36)		1 cat	12 cats 4 dogs	15 cats 3 dogs	1 cat	5 (2 to 7)
Cystostomy tube (*n* = 18)			1 cat	13 cats 3 dog	1 cat	15 (5 to 58)

#### Kidney rupture

Two dogs with ruptured kidneys secondary to trauma showed no azotaemia and were managed without any urinary diversion. One cat with urine leakage from a pyelogram site was managed with a peritoneal drain that had been placed intraoperatively during ureteral reimplantation. The drain was maintained for 7 days, and ultrasonography confirmed resolution of the peritoneal effusion.

#### Ureteral rupture

Two cases developed ureteral rupture post ureteral stent placement. Both cases were managed with peritoneal drains that had been placed intraoperatively, and a urethral catheter was also placed in one case. The drains and urethral catheter were removed after 2 days in both cases.

#### Urinary bladder rupture

Sixteen out of 19 cases of urinary bladder rupture were managed with urethral catheters. Additionally, abdominocentesis was performed in three of these cases, and a percutaneous wire‐guided peritoneal drain was placed in five of these cases. For the remaining 3 out of 19 cases of bladder rupture, urethral catheterisation was unsuccessful in two cats and not attempted in the third cat. These three cases were managed with peritoneal drains, combined with cystostomy tube placement in one cat. For all bladder ruptures, urethral catheters and peritoneal drains were left indwelling for a median of 3.5 days (range, 2 to 6 days) and a median of 2 days (range, 1 to 5 days), respectively.

#### Urethral rupture

Eight out of 25 cases of urethral rupture were managed with urethral catheters alone, six cases with cystostomy tubes alone and ten cases with a combination of urethral catheters and cystostomy tubes. One cat with a small urethral tear distal to the ischiatic tuberosity, which developed post‐urethral catheterisation, was managed without any urinary diversion due to the small size and location of the tear. A repeat retrograde urethrocystogram performed 12 days later showed no further contrast leakage, consistent with a healed tear.

Retrograde urethral catheterisation was attempted in 22 out of 25 cases. This was successful in eight cases, but failed initially in 14 cases, prompting attempts at other methods of urinary diversion (*e.g*. cystostomy tube placement). Retrograde urethral catheterisation was subsequently successful in three of these cases on repeat attempts.

Fifteen of the 16 cystostomy tubes were surgically placed, with only one placed percutaneously using a pigtail catheter. Eleven of the 18 urethral catheters were placed retrograde and seven were placed surgically using a hydrophilic weasel wire placed antegrade from the bladder through to the urethra *via* a cystotomy incision as a guidewire. For all urethral ruptures, urethral catheters and cystostomy tubes were left indwelling for a median of 6 days (range, 2 to 7 days) and a median of 14 days (range, 5 to 58 days), respectively.

### Resolution of urinary tract rupture

Resolution of urinary tract rupture was determined based on clinical signs in 14 cases and by imaging in 38 cases. The median time to resolution of urinary tract rupture as documented by imaging was 6 (range, 1 to 28) days (*n* = 37). Specifically, for bladder ruptures, the median time to documented resolution of rupture on imaging was 3 days overall (range, 1 to 6 days; *n* = 12), 5 days when managed with urethral catheters alone (range, 1 to 6 days; *n* = 7) and 2 days when managed with both urethral catheters and peritoneal drains (range, 1 to 3 days; *n* = 3). For urethral ruptures, the median time to documented resolution of rupture on imaging was 6.5 days overall (range, 3 to 28 days; *n* = 22), 6 days when managed with urethral catheters alone (range, 3 to 7 days; *n* = 6), 11 days when managed with cystostomy tubes alone (range, 4 to 21 days; *n* = 5) and 6.5 days when managed with both cystostomy tubes and urethral catheters (range, 5 to 38 days; *n* = 10).

### Outcome and complications associated with urinary diversion

The median duration of hospitalisation was 7 (range, 2 to 18) days from the time of rupture (*n* = 52). The median duration of follow‐up was 22.5 (range, 5 to 1049) days. Of the 25 cases of urethral ruptures, 16 cases continued to display lower urinary tract signs (such as stranguria and dysuria) after resolution of urinary tract rupture, six cases did not have any further associated signs, while the remaining three cases had urine drained regularly *via* their cystostomy tubes and there were no notes on voluntary urination.

Of the 16 cases with persistent lower urinary tract signs, eight cases had evidence of stricture or narrowing of the urethra on positive contrast radiographs, of which six underwent urethrostomy and two were euthanased as surgical management was declined (Table 3). For the three cases with no notes on voluntary urination, marked urethral narrowing was noted on positive contrast urethrocystography and all three cases underwent urethrostomy. Overall, the median time to radiographic diagnosis of stricture formation was 12 (range, 4 to 28) days post‐rupture, and the median time to urethrostomy was 13 (range, 6 to 66) days post‐rupture.

**Table 3:** Clinical findings and management of cases that developed urethral strictures

Case	Species	Age (m)	Sex/neuter status	Cause of rupture	Location of tear	Method of urinary diversion	Time from rupture to stricture (days)	Outcome (days post‐rupture)
1	Cat	20	MN	UO and catheterisation	Penile urethra	Cystostomy tube	4	PU (6)
2	Cat	65	MN	UO and catheterisation	Penile urethra	Cystostomy tube	11	PU (13)
3	Cat	111	MN	UO and catheterisation	Pelvic urethra and penile urethra	Urethral catheter and cystostomy tube	5	PU (16)
4	Cat	108	MN	UO and catheterisation	Junction of penile and pelvic urethra	Cystostomy tube	21^a^	TPU (66)
5	Cat	25	MN	External trauma	Pelvic urethra	Urethral catheter and cystostomy tube	28	TPU (41)
6	Cat	60	MN	External trauma	Pelvic urethra	Urethral catheter and cystostomy tube	23	PPU (23)
7	Cat	154	MN	External trauma	Pelvic urethra	Urethral catheter	13	PPU (13)
8	Cat	37	MN	UO and catheterisation	Pelvic urethra	Urethral catheter	6	Euthanasia
9	Dog	6	MN	UO and catheterisation	Penile urethra	Cystostomy tube	13	SU (14)
10	Dog	5	MN	Others	Penile urethra and pelvic urethra	Urethral catheter and cystostomy tube	10	SU (11)
11	Dog	199	ME	External trauma	Pelvic urethra	Urethral catheter	12	Euthanasia

ME Male entire, MN Male neutered, PPU Prepubic urethrostomy, PU Perineal urethrostomy, SU Scrotal urethrostomy, TPU Transpelvic urethrostomy, UO Urethral obstruction

^a^ At 21 days post‐rupture, moderate focal narrowing of the urethra was noted, but the urethra was still able to distend to a normal diameter with increased filling with contrast. At 62 days post‐rupture, only a narrow urethral catheter could be passed, and a tight narrowing was noted at the junction between the pelvic and penile urethra during catheterisation.

Excluding cases with pre‐existing urinary tract infections, bacterial culture of urine was performed in 22 patients and yielded bacterial growth in 14 cases, including 12 of 15 cases of urethral tears, one of five bladder tears and one of one ureteral rupture. The methods of urinary diversion in these 14 cases included urethral catheter (7/14), cystostomy tube (4/14) and urethral catheter combined with cystostomy tube (3/14). *Escherichia coli* was the most common isolate in 7/14 cases. Of the 11 patients with urethral strictures, seven had a positive culture associated with their urethral catheter or cystostomy tube, one had a pre‐existing pyelonephritis (diagnosed based on a positive culture from its subcutaneous ureteral bypass system and accompanying azotaemia) and the remaining three did not have any urine cultures performed.

## DISCUSSION

This study documents both iatrogenic and traumatic urinary tract ruptures being successfully managed conservatively. Conservative management was successful for ruptures along the entire urinary tract from the kidneys to the urethra, including in azotaemic and hyperkalaemic patients. Conservative management options are generally less invasive, and fast and simple to perform with readily available equipment; therefore, they may be particularly valuable in emergency and low‐cost settings. However, the cost associated with longer hospitalisation times for conservative management, compared to surgical management, should also be considered.

The aetiology of the rupture is likely to affect both the feasibility of conservative management and the tendency for conservative management to be considered by clinicians. Previous studies have reported that external trauma contributes to 30% to 56% of uroabdomen cases (Aumann et al., 1998; Grimes et al., 2018; Hornsey et al., 2021), but trauma only accounted for 8/52 (15.4%) cases of urinary tract ruptures in this study. Although bladder ruptures accounted for 19/52 sites of rupture in our study, none of them were caused by external (*e.g*. vehicular) trauma; they were predominantly induced iatrogenically by cystocenteses. This may reflect excluded trauma cases that failed medical management or no attempt at medical management based on extensive damage being considered likely in such cases. The skew towards iatrogenic causes may be because iatrogenic tears are perceived to be more confined, self‐resolving and amenable to conservative management. It is also interesting to note that a large proportion of bladder tears caused by cystocentesis had concurrent urethral obstruction; therefore, extra caution should be taken when performing cystocentesis in such patients.

This study illustrates the feasibility of conservative management, and its results may help generate hypotheses for future studies. For instance, the suitability of traumatic bladder ruptures for conservative management has not been previously evaluated. One experimental study found that three of 14 dogs with surgically induced bladder ruptures developed spontaneous sealing and healing of 3 cm incisions within their bladders without any treatment (Burrows & Bovee, 1974), so conservative management may have further scope than this study is able to document. The lack of traumatic bladder ruptures in this study likely represents clinician preference rather than failed management, but this cannot be confirmed with these data. Identification of criteria to dictate conservative *versus* surgical management, or consensus opinions, could be extremely useful.

Bladder ruptures were most commonly managed with urethral catheters and/or peritoneal drains, while urethral ruptures were most commonly managed with urethral catheters and/or cystostomy tubes. Cystostomy tubes were uncommonly used for bladder ruptures, likely because these cases would have undergone surgical repair of the bladder rupture if a surgical approach was taken. The median duration of urinary diversion ranged from 2 to 15 days, depending on the method used, while the median time to resolution of urine leakage, as documented on imaging, was 3 days for bladder ruptures and 6.5 days for urethral ruptures. Previous studies have reported similar median durations of urinary diversion ranging between 2.5 and 8.6 days (Addison et al., 2014; Anderson et al., 2006; Meige et al., 2008), although these were not differentiated based on the site of rupture, and resolution of leakage was not documented on imaging.

In this study, most peritoneal drains (9/12) were placed *via* a minimally invasive percutaneous approach, while most cystostomy tubes (17/18) were placed surgically. The reason for this disparity is unclear based on the available clinical records. One possible reason is the perceived risk of associated complications. The use of small‐bore wire‐guided catheters placed percutaneously for peritoneal drainage has been reported to have infrequent and minor adverse events, such as impaired drainage and fluid leakage around the insertion site (Crosby et al., 2023). In contrast, the percutaneous placement of pigtail catheters as cystostomy tubes has a reported complication rate of 40%, including complications such as bladder rupture and dislodgement of the catheter with secondary uroabdomen (Nurra et al., 2022). Other possible reasons include clinician preference or experience and logistical reasons (*e.g*. pigtail catheters not being readily available).

Eleven of 25 dogs and cats with urethral tears managed conservatively had radiographic evidence of urethral narrowing at a median of 12 days post‐rupture. In comparison, in a previous study evaluating 11 cats conservatively managed for urethral tears (Meige et al., 2008), narrowing was seen radiographically at the time of catheter removal in three cats, but only two cats became symptomatic, and only one underwent a prepubic urethrostomy at 6 weeks post‐rupture. In another study (Anderson et al., 2006), of 13 cats with urethral ruptures that were successfully managed conservatively and 11 cats that were successfully managed surgically, no strictures were reported, and of ten dogs undergoing primary urethral repair, no strictures were reported but two were euthanased for unknown reasons and detailed follow‐up was not available.

The cause of the relatively high rate of urethral narrowing in this study is unclear. Exposure to urine delays healing and promotes fibrosis, so it is commonly recommended to divert urine away from the site of injury to reduce the risk of stricture formation (Addison et al., 2014; Layton et al., 1987; Stafford & Bartges, 2013). Although urinary diversion was provided in all these cases, extraluminal exposure to urine (*via* urine within the peritoneal cavity, retroperitoneal cavity or subcutaneous tissues) could still promote stricture formation. Urinary tract infection may also contribute to stricture formation, as infection has been suggested to promote this condition (Degner & Walshaw, 1996). All these cases with strictures had a positive urine culture during or within 48 hours of the period of urinary diversion. Positive urine cultures are common in cats with complicated urethral obstructions requiring surgery (Corgozinho et al., 2007; Dumartinet et al., 2022), and urethral tears are also more likely in cats with complicated urethral obstructions (Manchester et al., 2024). This may suggest an association between infection, urethral tears and urethral strictures. Apart from the potential role of ongoing exposure to urine and urinary tract infections, the relatively early performance of urethrostomy in this study could also be due to premature diagnosis.

Studies have reported that between 8% and 48% of catheterised dogs develop urinary tract infections (Bubenik et al., 2007; Ogeer‐Gyles et al., 2006; Smarick et al., 2004), and 21/24 (88%) patients with cystostomy tubes had a positive urine culture while their cystostomy tube was in place (Beck et al., 2007). In comparison, 14/22 (64%) patients in this study had a positive urine culture associated with the urethral catheter or cystostomy tube. The relatively high prevalence of positive urine cultures in these patients highlights the importance of strict aseptic technique in maintaining urinary catheters and, more broadly, the implementation of local infection control measures. It has been suggested that the odds of urinary tract infection in dogs increase by 27% for each day of catheterisation (Bubenik et al., 2007). When indwelling urinary devices are employed in cases of urinary tract rupture, a risk/benefit analysis over the duration of catheterisation would therefore be recommended.

One limitation of this study is the small number of cases available, particularly for kidney and ureteral tears. Another limitation is the retrospective nature of the study. This study purely describes cases of urinary tract rupture that were successfully managed conservatively, and it was not possible to retrospectively determine why conservative management was elected over surgical management from the outset in these cases. This study design was chosen because it would not be possible to accurately distinguish between patients who had failed initial conservative management, thereby needing surgery, and patients who were successfully stabilized with conservative management prior to elective surgery. Other limitations include not having definitive confirmation of urinary tract rupture in two cases and not having follow‐up imaging to confirm resolution of urinary tract rupture in all cases. Future studies could aim to prospectively evaluate larger case numbers and more thoroughly compare different conservative management options for different sites of rupture.

In conclusion, iatrogenic and traumatic urinary tract ruptures can be successfully managed conservatively. Methods of conservative management include urethral catheters, peritoneal drains and cystostomy tubes; these can be placed *via* minimally invasive methods as appropriate. The duration of urinary diversion should be weighed against the risk of urinary tract infections. Of 25 animals with urethral tears, 11 had urethral narrowing documented and nine underwent urethrostomy procedures, so urethral stricture formation should be anticipated when opting for conservative management.

### Author contributions

**C. S. L. Toh:** Data curation (equal); formal analysis (equal); investigation (equal); methodology (equal); writing – original draft (lead). **M. Rossanese:** Conceptualization (equal); methodology (equal); validation (equal); writing – review and editing (equal). **S. D. Cook:** Conceptualization (equal); data curation (equal); formal analysis (equal); investigation (equal); methodology (equal); supervision (lead); validation (equal); writing – review and editing (equal).

### Conflict of interest

No conflicts of interest have been declared.

## Data Availability

The data that support the findings of this study are available from the corresponding author upon request.
